# The effects of whole-body muscle stimulation on body composition and strength parameters: A PRISMA systematic review and meta-analysis

**DOI:** 10.1097/MD.0000000000032668

**Published:** 2023-02-22

**Authors:** Luiz Rodrigues-Santana, Louro Hugo, Jorge Pérez-Gómez, Miguel A. Hernández-Mocholí, Jorge Carlos-Vivas, Pilar Saldaña-Cortés, Nicolás Contreras-Barraza, José C. Adsuar

**Affiliations:** a Faculty of Sport Science, University of Extremadura, Cáceres, Spain; b Sport Sciences School of Rio Maior, Research Center in Sport Science, Health and Human Development, Vila Real, Portugal; c Health, Economy, Motricity and Education (HEME), Faculty of Sport Science, University of Extremadura, Cáceres, Spain; d Physical Activity for Education, Performance and Health (PAEPH), Faculty of Sport Science, University of Extremadura, Cáceres, Spain; e Formación General, Universidad Autónoma de Chile, Santiago, Chile; f Facultad de Economía y Negocios, Universidad Andres Bello, Santiago, Chile; g Promoting a Healthy Society (PHeSo), Faculty of Sport Science, University of Extremadura, Cáceres, Spain.

**Keywords:** body composition, electromyostimulation, fat mass, lean body mass, strength, WB-EMS

## Abstract

**Background:**

This systematic review and meta-analysis set out to determine the efficacy of whole-body muscle electrostimulation on body composition, strength, and muscle power in active and non-active adults (aged ≥18 years).

**Method:**

This review was reported in accordance with the Protocol Statement of Preferred Reporting Element Guidelines for Systematic Reviews and Meta-Analysis included controlled trials; whole-body electromyostimulation trials with at least 1 exercise and control group; participants >18 years old. Outcome measures were defined as standardized mean differences for muscle mass, body fat mass, strength, and power. Studies were searched in the following electronic databases: PubMed, Web of Science, Scopus, SPORTDiscus, and EMBASE for all articles published up to July 30, 2021. The risk of bias was assessed by 2 independent researchers using the Physiotherapy Evidence Database scale and Grading of Recommendations, Assessment, Development and Evaluations approach. Analyses were performed using the metafor package of the statistical software R (version 4.0.3; R Core Team, 2020). Random effects models, forest, and funnel plots to quantify the asymmetry associated with publication bias were fitted using the metafor library in R. Statistical heterogeneity was assessed using *I*^2^ statistics.

**Results:**

In total, 26 studies representing 1183 participants were included (WB-electromyostimulation: n = 586 and control group: n = 597). The mean age of the participants ranged from a minimum of 20.4 to a maximum of 77.4 years old. Interventions lasted a minimum of 4 and a maximum of 54 weeks. Standardized mean difference was 0.36 (95% confidence interval [CI]: 0.16–0.57) for muscle mass, *−*0.38 (95% CI: −0.62–0.15) for body fat, 0.54 (95% CI: 0.35–0.72) for strength, and 0.36 (95% CI: 0.02–0.71) for power with significant differences between groups (all *P* < .04). *I*^2^ revealed low heterogeneity of muscle mass (15%) and power (0%) between trials and medium heterogeneity of body fat (45%) and strength (55%).

**Conclusion:**

We concluded that WB-electromyostimulation has significant positive effects on muscle mass, body fat, strength, and power.

## 1. Introduction

It is common knowledge that physical exercise is essential for a healthy lifestyle. The American College of Sports and Medicine recommends regular cardiovascular physical activity of 150 minutes a week with 2 sessions of muscular resistance training of major muscle groups, for the improvement and maintenance of cardiorespiratory, musculoskeletal, and neuromotor fitness in apparently healthy individuals.^[1,2]^

Body composition is one of the main indicators of physical health and well-being. In fact, changes in body composition throughout life are related to mortality risk.^[3]^ According to the American Council on Exercise,^[4]^ the healthy fat percentage for adults is up to 24% for men and 31% for women, with higher values considered as excess body fat, which is the main cause of obesity and other metabolic and cardiovascular diseases. In addition, the amount of muscle mass and strength play an important role, since as we age, muscle mass tends to decrease and its loss is directly related to a decrease in functional capacity, poorer quality of life, and dependence in older people.^[5]^ In recent years, new approaches have emerged with the premise of shorter and more efficient workouts. One example is high-intensity interval training programs, which have a positive impact on fat loss and muscle mass gain,^[6]^ and is nowadays one of the most widely used strategies to improve body composition, as this training method has shown similar results to those obtained after applying a traditional continuous training program of moderate intensity, with 40% less duration.^[7]^ Another recent approach using technology is whole-body electrostimulation, a time-saving training method used worldwide. Its use has increased in recent years among the population seeking faster results in less time.^[8]^ The training programs are variable according to the objectives and characteristics of its practitioners, increasing their physical condition and improving body composition with the most outstanding benefits, according to experts and manufacturers.^[9–11]^ Previous studies^[12–15]^ have proven the effectiveness of whole-body electromyostimulation (WB-EMS) and its use as an alternative sporting activity, both for those fleeing from conventional methodologies and for athletes who wish to improve their sporting performance through WB-EMS sessions. This training method has also demonstrated improvements in body composition and strength in older people^[16–20]^ and in active and healthy populations.^[21,22]^ The main users of this methodology are middle-aged women, who perform 2 workouts a week in order to lose weight, improve health, and gain muscle mass.^[23]^

Although there is the possibility of using very diverse protocols, in the current literature the most common is the application of bipolar stimuli as more usual, with a period of stimulation and another of pause (intermittent) with a frequency of 50 to 80 Hz and depth of 300/400 µs. The average duration of this type of training is 20 minutes.^[24]^

Given the number of randomized controlled trials in different types of populations seeking to determine the effectiveness of WB-EMS on body composition and strength parameters, in order to improve our knowledge of the use of WB-EMS and its effects we conducted a systematic review and meta-analysis of published studies associating these 2 variables. The primary objective of this study is to determine the efficacy of WB-EMS for composition improvement and secondly to evaluate the effects of this training on some strength parameters. Thus, our primary hypothesis was that WB-EMS enhances the positive effects on lean body mass and fat mass loss. Furthermore, our secondary hypothesis was that WB-EMS generates positive effects on strength and muscle power.

## 2. Materials and methods

### 2.1. Literature search and study selection

Studies were searched in the following electronic databases: PubMed, Web of Science, Scopus, SPORTDiscus, and EMBASE for all articles published until July 30, 2021, in the English language only, with no publication status limitations. All randomized clinical trials will be considered. Details of the Cochrane Library are presented in Table 1. It was registered in advance in INPLASY (INPLASY202120050). This review was reported in accordance with the Protocol Statement of Preferred Reporting Element Guidelines for Systematic Reviews and Meta-Analysis.^[25]^

**Table 1:** Search terms used in literature search.

Category 1	Category 2	Category 3
Whole Body Electro muscle stimulation WB-EMS Whole-body-electro-myo-stimulation Whole-body Electromyostimulation Whole-Body Electromyostimulation training Neuromuscular Electrical Stimulation NMES	Body Composition Fat Mass OR/AND Muscle Mass Strength OR/AND Power	Randomized controlled trial Controlled trial Clinical trial

WB-EMS = whole-body electromyostimulation.

### 2.2. Eligibility criteria

#### 2.2.1. Study types.

This study included randomized clinical trials investigating the effects of whole-body electrostimulation training on body composition and strength indicators.

#### 2.2.2. Intervention types.

In the intervention group, all subjects must have performed the same exercise protocol with the full-body electrostimulation suit. In the control group (CG), participants must not have performed any training program.

#### 2.2.3. Participant types.

All studies involving trained or untrained participants >18 years of age, with no previous experience with WB-EMS, will be considered.

#### 2.2.4. Outcome measurements.

The primary outcomes of the study are fat-free mass or muscle mass and percentage fat mass or amount of fat measured by electrical bioimpedance, dual-energy X-ray absorptiometry, skinfolds, or anthropometric measurements. Secondary outcomes shall be the maximum strength and muscle power measured in different tests.

### 2.3. Study selection

**Figure 1.**
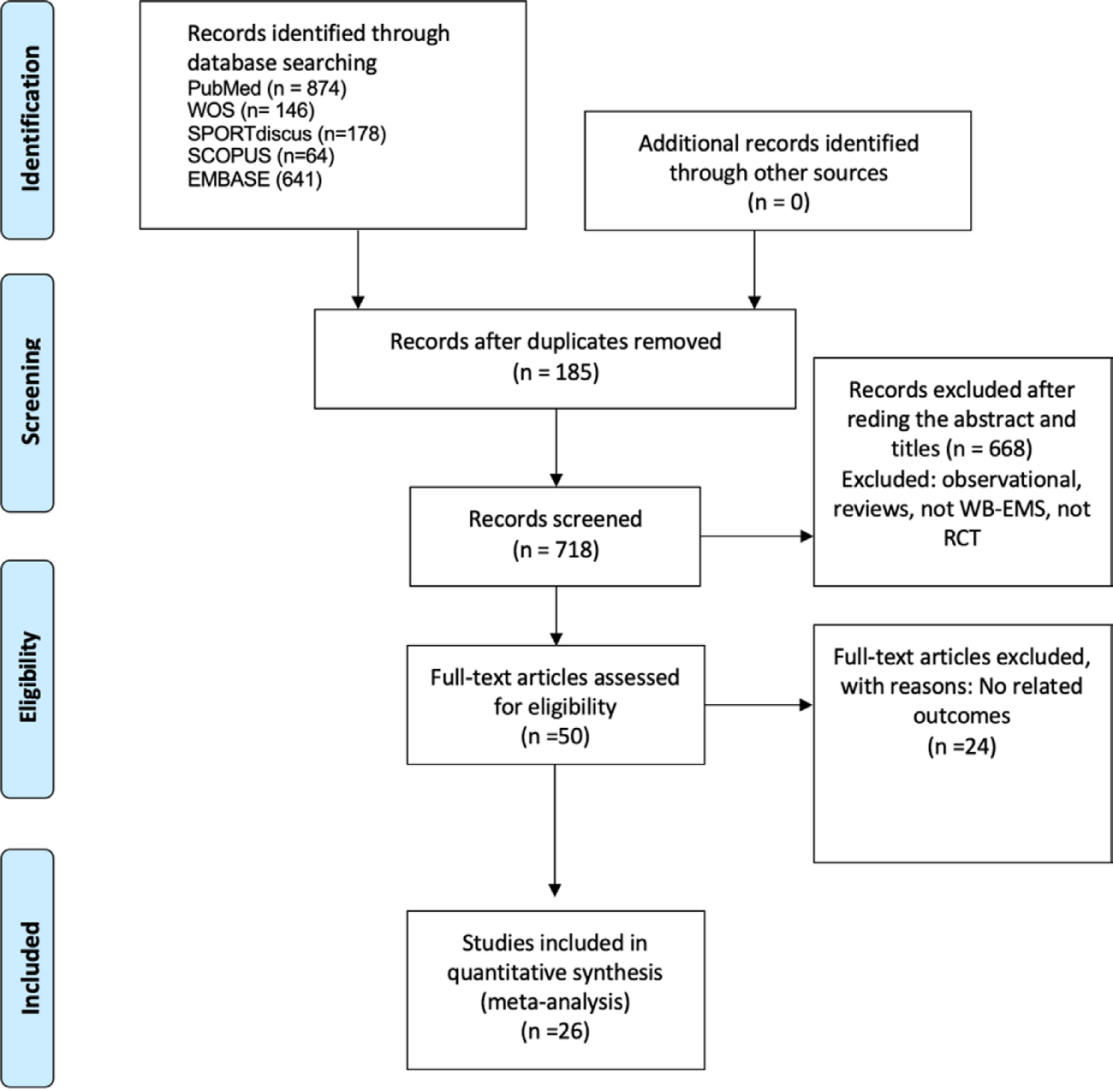
Flow diagram of search process.

Two authors independently screened study titles/abstracts and excluded unrelated studies. They then read the full articles of the remaining studies according to the eligibility criteria. The study selection process is shown in a flowchart according to the PRISMA guidelines (Fig. 1).

### 2.4. Data collection and management

Two authors independently collected data from all studies based on the data extraction form. This consisted of collecting the following information: title, authors, study design, participant characteristics, type of treatments and controls, outcomes, and other essential data elements. Any discrepancies were discussed with an experienced third author by discussion.

### 2.5. Missing data dealing with

Once we identified missing or unclear data, we contacted the original authors of the trial to request it. One author responded to our queries.^[26]^

### 2.6. Risk of bias assessment

The risk of bias was assessed by 2 independent researchers using the Physiotherapy Evidence Database (PEDro) scale.^[27]^ The PEDro scale consists of 11 items, of which only 10 (0/1) are scored. The PEDro scale refers to randomization, allocation concealment, similarity at baseline, blinding of participants, staff and assessors, incomplete outcome data, intention-to-treat analysis, between-group comparison, and measure of variability. In addition, the Grading of Recommendations, Assessment, Development and Evaluations (GRADE) system^[28]^ will be used to rank the quality of evidence and the strength of the recommendation. The GRADE approach to assessing the quality of evidence involves a 4-point scale including “high,” “moderate,” “low,” and “very low.” It started at the high level and was downgraded to lower levels of evidence when there was a risk of bias, inconsistency, indirectness, imprecision, or publication bias. In addition, the GRADE system^[28]^ will be used to rank the quality of evidence and strength of recommendation.

### 2.7. Data synthesis

Results from different studies have been organized in the same way, as effect sizes and corresponding standard errors. The changes in means and standard deviations, as well as sample size, for each group of different studies, have been used to calculate effect sizes as Hodges g using the library esc in the R statistical software (version 4.0.3; R Core Team, 2020; https://cran.r-project.org/web/packages/metafor/index.html).^[29]^ These effect sizes represent the changes between study groups over the intervention time.

For studies that reported their results as means and standard deviations in pre-post format, changes for both were computed using the following formula as suggested by Higgins (2011)^[30]^:


$$\begin{array}{l} Mean_{diff}=Mean_{post}-Mean_{pre} \end{array}$$

$$\begin{array}{l} SD_{diff}=\sqrt{SD_{pre}^{2}+SD_{post}^{2}-(2*0,5*SD_{pre}*SD_{post}} \end{array}$$

Some studies reported its results as means and interval confidence of changes. For that scenario, using the same reference as previously, standard deviations changes were calculated as


$$\begin{array}{l} SD_{diff}=\frac{\sqrt{N}*SDdif_{UL}-SDdif_{LL}}{3,92} \end{array}$$

### 2.8. Statistical analysis

The analyses were conducted using the metafor package of the statistical software R (version 4.0.3; R Core Team, 2020). Random effects models, forest, and funnel plots^[31]^ for quantifying asymmetry associated with publication bias were adjusted by means of metafor library in R.^[32]^ Statistical heterogeneity was assessed using *I*^2^ statistics (low: 0–39%, moderate: 40–59%, substantial: >60%.^[33]^

### 2.9. Ethics and dissemination

Ethical approval is not required as individual patient data will not be collected in this study. We will publish this study in a peer-reviewed journal.

## 3. Results

Full descriptive details of the included studies are shown in Table 2. Twenty-six studies were included in this systematic review and meta-analysis, with a total of 1183 subjects (randomized control trial: n = 586, CG: n = 597). The range of participants in each group varied from n = 8^[15]^ to n = 55^[34]^ in the WB-EMS group and in the CG. The mean age of the participants ranged from a minimum of 20.4 (electrostimulation exercise group [EEG] group) and 20.5 (CG)^[35]^ to 77.3 (EEG group) and 77.4 (CG).^[36]^ Interventions lasted a minimum of 4 weeks^[35]^ and a maximum of 54 weeks.^[37]^

**Table 2:** Assessment of risk of bias for included studies (n = 27) according to PEDro scale.

References	Eligibility criteria	Random allocation	Allocation concealment	Inter group homogeneity	Blinding subjects	Blinding personnel	Blinding assessors	Participation ≥85% allocation	Intention to treat analysis	Between group comparison	Measure of variability	Total score PEDro
Alvaro Pano-Rodrigues et al (2020)	Y	1	1	1	0	1	1	1	1	1	1	9
Alvaro Pano-Rodrigues et al (2020b)	Y	1	1	1	1	0	1	1	1	1	1	9
Andre Filipovic et al (2019)	Y	1	1	1	0	0	0	1	1	1	1	7
Anja Weissenfels (2018)	Y	1	1	1	0	0	1	1	1	1	1	8
Anja Weissenfels (2019)	Y	1	1	1	0	0	1	1	1	1	1	8
Berger, J (2020)	Y	1	1	1	1	1	0	1	1	1	1	9
Carina Zink-Ruckel et al (2021)	Y	1	1	1	1	0	0	1	1	1	1	8
Evangelista A, et al (2019)	Y	1	1	1	0	0	1	1	1	1	1	8
Evangelista A, et al (2021)	Y	1	1	1	0	0	0	1	1	1	1	7
Florian Mick (2018)	Y	1	1	1	0	0	0	0	1	1	1	6
Francisco J. Amaro-Gahete et al (2018)	Y	1	1	1	0	0	0	0	1	1	1	6
Francisco J. Amaro-Gahete et al (2018b)	Y	1	1	1	0	0	1	0	1	1	1	7
Hyeng-Kyu Park et al (2020)	Y	1	1	1	1	1	1	1	1	1	1	10
Jiyoun Kim et al (2020)	Y	1	0	1	1	0	0	1	1	1	1	7
Katharina Wittmann et al (2016)	Y	1	1	1	0	0	1	1	1	1	1	8
Sebastian Willert et al (2019)	Y	1	1	1	0	0	1	1	1	1	1	7
Simon von Stengel (2015)	Y	1	1	1	1	0	0	1	1	1	1	8
Stefano D’ottavio (2019)	Y	1	1	1	0	0	0	1	1	1	1	7
Sunhee Park et al (2021)	Y	1	1	1	1	0	0	1	1	1	1	8
L. Jurado-Fasioli et al (2019)	Y	1	1	1	0	0	1	1	1	1	1	8
Ulrike Dörmann (2019)	Y	1	1	1	1	0	0	0	1	1	1	7
Wolfgang Kemmler et al (2010)	Y	1	0	0	0	0	1	1	1	1	1	6
Wolfgang Kemmler et al (2013)	Y	1	1	1	1	0	1	0	1	1	1	8
Wolfgang Kemmler et al (2016)	Y	1	1	1	0	0	1	1	1	1	1	8
Wolfgang Kemmler et al (2018a)	Y	1	1	1	0	0	1	0	1	1	1	7
Wolfgang Kemmler et al (2018b)	Y	1	1	1	0	0	1	1	1	1	1	8
Yong-Seok Jee (2019)	Y	1	0	1	1	0	0	1	0	1	1	6

PEDro = Physiotherapy Evidence Database.

### 3.1. Characteristics of the studies and participants

In relation to the training volume of the intervention groups, there was a variation of 1 training per week and a maximum of 3^[38–42]^ with a duration between 8^[13]^ and 40 minutes.^[39]^ Among the 26 studies included, 11 have been conducted with women only^[16,20,35–39,42–45]^ and 7 were only with men.^[13,26,41,46–50]^ Two interventions have been performed in patients with chronic low back pain,^[34,51]^ 4 in people with sarcopenia and obese,^[20,39,46,52]^ 4 in pre- and post-menopausal women^[43–45,53]^ and 1 in older people with osteoarthritis.^[38]^ Two studies have worked with athletes.^[13,15]^ All other studies have been conducted in healthy, untrained, or inactive people.

### 3.2. Type of interventions and groups

Most studies have isolated the application of WB-EMS in the intervention group, while 2 studies added protein supplementation^[26,36]^ and another study restricted energy intake.^[45]^ In addition, in 2 other interventions, the authors included groups with different stimulus frequencies (Hz).^[54,55]^ A single article worked with groups with different stimulus intensities.^[41]^

The training comparison groups were performed with the same exercise program as the WB-EMS group, with the exception of 1 study where the WB-EMS was compared with a CG without any exercise program ^[51]^ and 4 other articles in which the comparison group performed other training programs parallel to the WB-EMS group.^[21,37,45,56]^

### 3.3. WB-EMS protocol used

The WB-EMS protocols (i.e., the pulse parameters) were fairly homogeneous across the studies. All studies applied low-frequency bipolar protocols of 80 to 85 Hz with a rectangular pulse waveform, with the exception of 4 studies that applied 20 Hz, 55 Hz, and 50 Hz.^[43,44,54,55]^ The pulse was specified between 200 and 400 ms. All studies combined WB-EMS with dynamic voluntary movements, with the exception of 3 studies that applied isometric exercises.^[38,41,54]^

The intensity of the application pulse has been predominantly prescribed according to Borg rating of perceived exertion,^[57]^ ranging from consistently strong^[5]^ to very strong^[7]^ on the 10-point scale and 15 to 19 on the 20-point scale. However, 5 working groups used a maximum impulse tolerance approach.^[35,38,39,48]^ and a single article worked the stimulus intensity between 80 and 100 mA.^[21]^ It remains important to note that none of the studies reported negative side effects of WB-EMS applications Table 3. Study and intervention characteristics of the included articles (n = 26).

**Table 3:** Study and intervention characteristics of the included articles (n = 26).

References	Sample size (n); population; AEG (mean ± SD)	Groups	Intervention	Mains outcomes
Alvaro Pano-Rodrigues et al (2020)	Postmenopausal women N = 32 61,38 + −3,95 yr old	WB-EMS = 16 e.g. = 16	10 wk. 2 sessions × 20 min per wk. WB-EMS group performed 20 repetitions of 3 exercises (squat, deadlift, and bench press). Stimulus of 55 Hz, 200–400 μs with RPE 15 (Borg Scale-20). e.g. performed the same exercise protocol as the WB-EMS group (without the EMS).	Body composition, power, and speed
Alvaro Pano-Rodrigues et al (2020b)	Postmenopausal women N = 34 61,4 + −4,0 yr old	WB-EMS = 17 e.g. = 17	10 wk. 2 sessions × 20 min per wk. WB-EMS group performed 20 repetitions of 3 exercises (squat, deadlift, and bench press). Stimulus of 55 Hz, 200–400 μs with RPE 15 (Borg Scale-20). e.g. performed the same exercise protocol as the WB-EMS group (without the EMS).	Balance, strength, flexibility, and agility
Andre Filipovic et al (2019)	Soccer players N = 28 WB-EMS = 24,4 + −4,2 yr old TG = 21,1 + −1,9 yr old CG = 23,6 + −3,9 yr old	WB-EMS = 10 e.g. = 10 CG = 8	7 wk. 2 sessions × 8 min per wk. WB-EMS group performed 3 × 10 jump squats. Stimulus of 85 Hz, 350 μs with RPE 16–19 (Borg Scale-20). e.g. performed the same exercise protocol as the WB-EMS group (without the EMS). CG did not exercise.	Body composition, muscle biopsy, and strength
Anja Weissenfels (2018)	Low back pain patients N = 30 WB-EMS = 54,6 + −5,7 CG = 59,4 + −7,7	WB-EMS = 15 CG = 15	12 wk. 1 session × 20 min per wk. WB-EMS group performed light movements to relieve back pain. Stimulus of 85 Hz, 350 μs with RPE 5–7 (Borg10 Scale). Control group did not exercise.	Low back pain intensity, isometric maximum force of extension, and flexion of the trunk
Anja Weissenfels (2019)	Low back pain patients N = 110 WB-EMS = 57,4 + −7,6 CG = 54,4 + −7,4	WB-EMS = 55 e.g. = 55	12 wk. 1 session × 12–20 min per wk. WB-EMS group performed 6 specific exercises for the trunk muscles. Stimulus of 85 Hz, 350 μs with RPE 5–7 (Borg Scale10). e.g. performed the same exercise protocol as the WB-EMS group (without the EMS)	Intensity of lumbar pain, maximum isometric strength of extension, and flexion of the trunk
Berger, J (2020)	N = 51 24,9 + −3,9	WB-EMS = 19 WB-EMS20 = 14 CG = 14	10 wk. 1.5 sessions × 20 min per wk. WB-EMS group performed 10–12 repetitions of 10 selected exercises. Stimulus of 20–85 Hz, 350 μs with RPE 6–7 (Borg Scale 10). e.g. performed the same exercise protocol as the WB-EMS group (without the EMS). Control group did not exercise.	Jump, speed, and strength
Carina Zink-Ruckel et al (2021)	Amateur golfers N = 54 WB-EMS = 42,7 + −16,6 yr old CG = 43,0 + −13,4 yr old	WB-EMS = 27 e.g. = 27	16 wk. 1 session of 20 min per wk. WB-EMS group performed 6–8 repetitions of light movements. Stimulus of 80 Hz, 350 μs with RPE 6–7 (Borg Scale-10). e.g. performed the same exercise protocol as the WB-EMS group (without the EMS)	Intermuscular adipose tissue and volume and interfacial muscle tissue
Evangelista A, et al (2019)	N = 58 WB-EMS = 25,5 + −6,1 e.g. = 25,1 + −3,2 CG = 27,1 + −4,1	WB-EMS = 21 e.g. = 21 GC = 16	8 wk. 2 sessions × 20 min per wk. WB-EMS group performed 3 sets of 8 to 12 repetitions maximum of 3 exercises. Stimulus of 80–85 Hz, 350 μs with RPE 5–8 (Brog Scale-10). e.g. performed the same exercise protocol as the WB-EMS group (without the EMS). Control group did not exercise.	Maximum strength and muscle thickness
Evangelista A, et al (2021)	Inactive older men N = 20 75,1 + −6,58 yr old	WB-EMS = 10 e.g. = 10	6 wk. 2 sessions × 20 min per wk. WB-EMS group performed 8 bodyweight exercises in 2 sets × 8 repetitions. Stimulus with 85 Hz, 350 μs with RPE 7–8 (Borg-10 Scale). e.g. performed the same exercise protocol as the WB-EMS group (without the EMS).	Body composition and physical condition
Florian Mick (2018)	Sport students N = 18 WB-EMS = 22,8 + −3,0 CG = 22,8 + −2,5	WB-EMS = 8 e.g. = 8	8 wk. 2 sessions × 20 min per wk. WB-EMS group performed 5 exercises (2 strength + 3 dynamic jump exercises). Stimulus with 85 Hz, 350 μs, and 70% of the maximum tolerance capacity. e.g. performed the same exercise protocol as the WB-EMS group (without the EMS).	Strength, power, jump, and speed
Francisco J. Amaro-Gahete et al (2018)	Middle-aged adult N = 65 3,5 + −4,9 yr old	WB-EMS = 19 e.g.=16 PAR = 16 GC = 14	12 wk. 2 sessions × 20–30 min per wk. WB-EMS group performed 2 different sessions: HIIT with long intervals and HIIT with short intervals. Stimulus with 15–75 Hz, 200–400 μs, and 80–100 mA of intensity. e.g. performed the same exercise protocol as the WB-EMS group (without the EMS). PAR performed the WHO-recommended exercise protocol 3 × per wk. Control group did not exercise.	Body composition, physical activity assessment, and dietary intake assessment
Francisco J. Amaro-Gahete et al (2018b)	Amateur runners N = 12 27,0 + −6,8 yr old	WB-EMS = 6 e.g. = 6	6 wk. 1 session × 16–20 min per wk. WB-EMS group performed wave periodization model The training sessions were divided into 4 parts: warm-up (phase A), strength training part (phase B), high-intensity interval power training part (phase C), and high-intensity training part. (Phase D). Stimulus of 12–90 Hz, 200–400 μs, and RPE 10–17 (Borg-10 scale). e.g. kept up his race training routine	VO2 max, running economy, muscle power, and body composition
Hyeng-Kyu Park et al (2020)	Young women N = 23 WB-EMS = 23,5 + −4,2 CG = 25,2 + −5,7	WB-EMS = 11 e.g. = 12	6 wk. 3 sessions × 20 min per wk. WB-EMS group performed low-intensity strength exercises. 80 Hz stimulus. e.g. performed the same exercise protocol as the WB-EMS group (without the EMS).	Body composition; laboratory lipid profile; magnetic resonance imaging; assessment of isokinetic muscle function; evaluation of balance function and cardiopulmonary function test
Jiyoun Kim et al (2020)	Obese older women N = 25 EC = 71,75 + −2783 CG = 70,38 + −2,93	WB-EMS = 13 e.g. = 12	8 wk. 3 sessions × 40 min per wk. WB-EMS group performed anaerobic and aerobic exercises with dance. Stimulus of 85 Hz, 350 μs, and intensity of 60–80% of the maximum tolerance capacity. e.g. performed the same exercise protocol as the WB-EMS group (without the EMS).	Body composition, biomarkers, and caloric intake/expenditure
Katharina Wittmann et al (2016)	Sarcopenic obese older women N = 67 EC = 77,3 + −4,9 yr old ECP = 76,4 + −2,9 yr old CG = 77,4 + −4,9 yr old	WB-EMS = 24 WB-EMSP = 21 CG = 22	26 wk. 1 session × 20 min per wk. WB-EMS group performed a guided video program in a sitting position with light movements for the lower and upper body. Stimulus with 85 Hz, 350 μs, and RPE 5–6 (Borg-10 Scale). e.g. performed the same exercise protocol as the WB-EMS + protein supplementation group. Control group did not exercise.	MetS Z-score, waist circumference; mean arterial pressure, triglycerides, fasting plasma glucose, and high-density lipoprotein cholesterol
Sebastian Willert et al (2019)	Overweight premenopausal women N = 90 EC = 38,4 + −8,0 yr old PA = 34,4 + −8,3 yr old CG = 35,3 + −7,4 yr old	WB-EMS = 30 PA = 30 CG = 30	16 wk. 1.5 sessions × 20 min per wk. WB-EMS group performed 2 sets of 6–8 repetitions of light exercises. Stimulus with 85 Hz, 350 μs, and RPE 5–7 (Brog-10 Scale). PA group increases their normal daily activity by 250 kcal/d and reduces energy intake by 250 kcal/d. CG reduced energy intake by 500 kcal/d.	Lean body mass and body fat mass
Simon von Stengel (2015)	Osteopenia women N = 60 WB-EMS = 74,7 + −3,7 CG = 74,7 + −4,4	WB-EMS = 32 e.g. = 28	54 wk. 1.5 sessions × 18–19 min per wk. WB-EMS group performed 10–14 exercises 1–2 sets of 8 repetitions. Stimulus with 85 Hz, 350 μs, and RPE 14–16 (Borg Scale-20). e.g. performed the same exercise protocol as the WB-EMS group (without the EMS).	Bone mineral density, lean body mass, and grip strength
Stefano D’ottavio (2019)	N = 22 26,7 + −3,15	WB-EMS = 6 WB-EMS50 = 8 e.g. = 8	6 wk. 2 sessions × 20 min per wk. WB-EMS group performed 9 isometric exercises. Stimulus with 50–85 Hz, 350 μs, and RPE 14–16 (Borg-20 Scale). WB-EMS50 performed the same exercise protocol with a 50 HZ stimulus. e.g. performed the same exercise protocol as the WB-EMS and WB-EMS50 groups (without the EMS).	Strength and power
Sunhee Park et al (2021)	Elderly women with knee osteoarthritis N = 75 EC = 65,68 + −3,24 ISO = 66,88 + −4,61 CON = 68,04 + −4,16	EC = 25 ISO = 25 GC = 25	8 wk. 3 sessions × 20 min per wk. WB-EMS group performed isometric exercises with the duration of the device contraction (6 s). Stimulus with 85 Hz, 350 μs, and 60–70% of maximum tolerance capacity. ISO e.g. performed the same exercise protocol as the WB-EMS group (without the EMS). CG performed meditation and gentle stretching.	Body composition, biomarkers, strength, and degree of knee injury and osteoarthritis
L. Jurado-Fasioli et al (2019)	Sedentary adults N = 67 53,3 + −5,0	WB-EMS = 17 HIIT = 18 PAR = 17 GC = 15	12 wk. 2 sessions × 20–30 min per wk. WB-EMS group performed 2 different types of training: HIIT with long breaks and HIIT with short breaks. Stimulus of 15–75 Hz, 200–400 μs, and 80–100 mA of intensity. HIIT group performed the same exercise protocol as the WB-EMS group (without EMS). The PAR group performed the exercise protocol recommended by the WHO. GC did not exercise.	Body composition and caloric intake
Ulrike Dörmann (2019)	Active women N = 22 20,5 + −2,3 yr old	EC = 11 e.g. = 11	4 wk. 2 sessions × 20 min per wk. WB-EMS group trained with different exercises. Session a) strength b) speed and jump exercises. Stimulus with 85 Hz, 350 μs, and 70% of the maximum tolerance capacity. e.g. performed the same exercise protocol as the WB-EMS group (without the EMS).	Jump and speed test Strength and power
Wolfgang Kemmler et al (2010)	Postmenopausal women N = 30 64,5+ −5,5 yr old	WB-EMS = 15 e.g. = 15	14 wk. 1.5 sessions × 20 min per wk. WB-EMS group performed 1–2 sets of 10 exercises with 6–8 repetitions and conventional group training. Stimulus with 85 Hz, 350 μs, and RPE 6–7 (Borg-10 Scale). e.g. only did the conventional workouts.	Body composition, strength, and metabolic rest rate
Wolfgang Kemmler et al (2013)	Older women N = 60 EC = 74,4 + −3,7 yr old CG = 74,7 + −4,4 yr old	WB-EMS = 32 WB-EMS = 28	54 wk. 1.5 sessions × 18 min per wk. WB-EMS group performed exercises of low intensity and amplitude. Stimulus with 85 Hz, 350 μs, and RPE 14–16 (Borg-20 Scale). WB-EMS performed 1 session × 60 min for 10 wk interspersed with 10 wk off during the 54 wk.	Body composition and strength
Wolfgang Kemmler et al (2016)	Untrained men N = 42 EC = 41,9 + −6,4 HIIT = 43,7 + −6,1	EC = 22 HIIT = 20	16 wk. 1.5 sessions × 20 min per wk. WB-EMS group performed 1–2 series of 12 exercises and 6–8 repetitions. Stimulus with 85 Hz, 350 μs, and RPE 6–7 (Borg-10 Scale). HIIT did 2–3 sessions a wk, 10–13 exercises. Two sets of 15 repetitions in high-intensity interspersed training	Body composition and strength
Wolfgang Kemmler et al (2018a)	Older men with sarcopenic obesity N = 100 EC = 77,1 + −4,3 PC = 78,1 + −5,1 CG = 76,9 + −5,1	WB-EMS = 33 WB-EMSP = 33 GC = 34	16 wks. 1.5 sessions × 20 min per week. WB-EMS group performed 1–2 series of 12 exercises and 6–8 repetitions. Stimulus with 85 Hz, 350 μs, and RPE 6–7 (Borg-10 Scale). e.g. performed the same exercise protocol as the WB-EMS group (without the EMS). PG was protein supplemented without exercising; GC did not exercise.	Body composition, sarcopenia Z-score, and grip strength
Wolfgang Kemmler et al (2018b)	Older men with sarcopenic obesity N = 67 77,0 + −5,0 yr old	WB-EMS = 33 e.g. = 34	16 weeks. 1.5 sessions × 20 min per week. WB-EMS group performed 1–2 series of 12 exercises and 6–8 repetitions. Stimulus with 85 Hz, 350 μs, and RPE 6–7 (Eschar Borg-10). e.g. performed the same exercise protocol as the WB-EMS group (without the EMS).	Body composition, strength, MetS, and kidney function
Yong-Seok Jee (2019)	Healthy college men N = 54 WB-EMS80hz = 25,00 + −0,82 WB-EMS60Hz = 23,25 + −2,22 WB-EMSHz50 = 24,00 + −3,00 e.g.=28,33 + −2,52	WB-EMS80Hz = 13 WB-EMS60Hz = 14 WB-EMS50Hz = 12 e.g. = 13	6 wk. 3 sessions × 20 min per wk. WB-EMS group performed isometric exercises, 12 repetitions during the impulse phase (6 s). Stimulus with 85 Hz, 350 μs, and 50%, 60%, and 80% of the maximum tolerance capacity. WB-EMS80 performed the exercises with an intensity of 80%; WB-EMS60 performed the exercises with an intensity of 60%; WB-EMS50 performed the exercises with an intensity of 50%. e.g. performed the same exercise protocol as the WB-EMS group (without the EMS).	Body composition, strength, and thigh circumference

CG = control group, EC = exercise group, EMS = electromyostimulation, HIIT = high intensive interval training group, PAR = physical activity recommendation group, RPE = rated perceived exertion, WB-EMS = whole-body electromyostimulation group.

### 3.4. Risk of bias assessment

Table 2 shows the risk of bias for included studies according to the PEDro scale. According to the PEDro scale, most of the studies have a high methodological quality (22 of 26 included articles). Four studies were rated as studies of moderate methodological quality.^[15,16,41,58]^

The quality of evidence according to the GRADE approach was “low,” meaning that “Further research is very likely to have a major impact on our confidence in the estimate of effect and is likely to change the estimate” or “Our confidence in the estimate effect is limited: The true effect may be substantially different from the estimate of the effect.”^[59,60]^

### 3.5. Effects of WB-EMS on muscle mass

**Figure 2.**
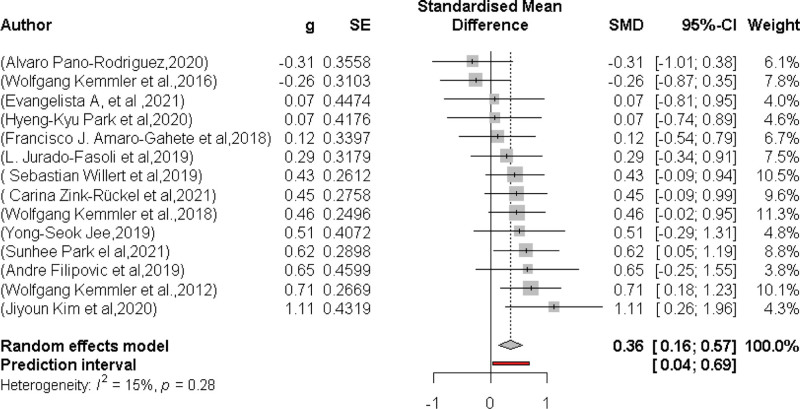
Forest plot of the results of the meta-analysis on muscle mass. Data are shown as pooled standardized mean differences (SMD) with 95% CI for changes in the WB-EMS and non-EMS training groups. CI = confidence interval, WB-EMS = whole-body electromyostimulation.

Fourteen studies with the same number of WB-EMS groups evaluated the effect of WB-EMS on muscle mass (Fig. 2). In summary, the WB-EMS intervention produced significant effects (*P* = .002). The standardized mean difference (SMD) between all groups was = 0.36; 95% confidence interval (CI): 0.16–0.57 with a low level of heterogeneity between trials (*I*^2^ = 15%).

**Figure 3.**
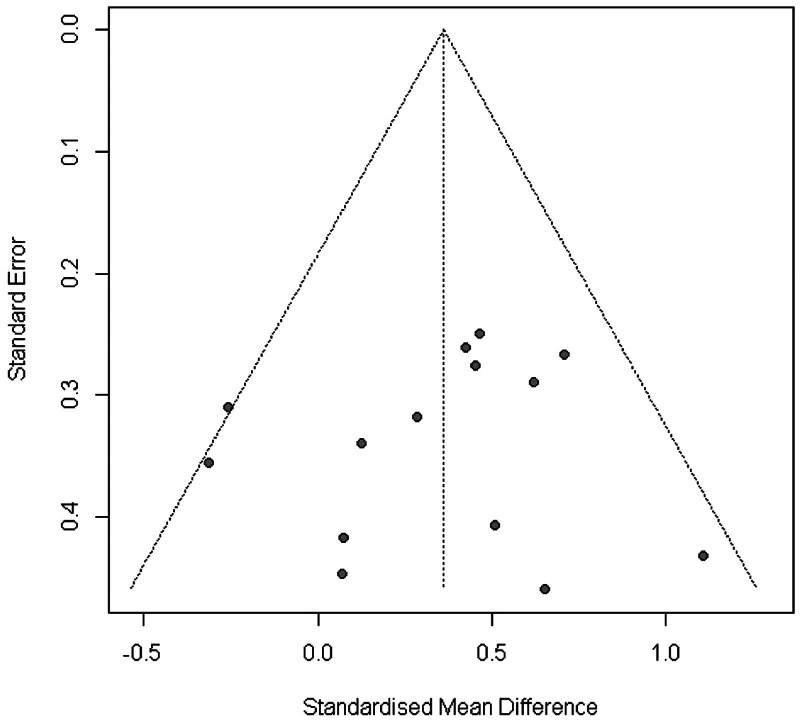
Funnel plot of WB-EMS studies addressing muscle mass. WB-EMS = whole-body electromyostimulation.

In summary, the Funnel plot (Fig. 3) provided evidence of small study bias.^[61]^ Egger regression test^[31]^ for funnel plot asymmetry did not indicate significant asymmetry (*P* = .7).

### 3.6. Effects of WB-EMS on body fat

**Figure 4.**
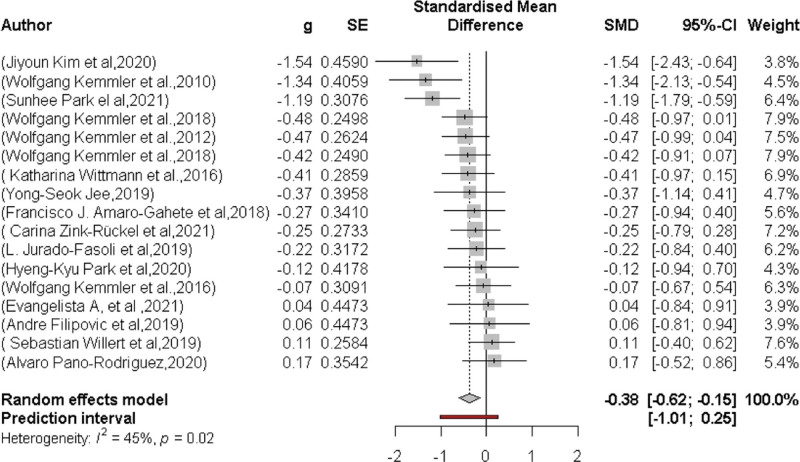
Forest plot of the results of the meta-analysis on body fat. Data are shown as pooled standardized mean differences (SMD) with 95% CI for changes in the WB-EMS and non-EMS training groups. CI = confidence interval, WB-EMS = whole-body electromyostimulation.

Seventeen studies with the same study groups determined the effect of EEG on total body fat mass (Fig. 4). In summary, EEG significantly (*P* = .003) affected total body fat mass. The SMD was −0.38 95% CI: −0.62–0.15. A moderate level of heterogeneity was observed between trials (*I*^2^ = 45%, *P* = .02).

**Figure 5.**
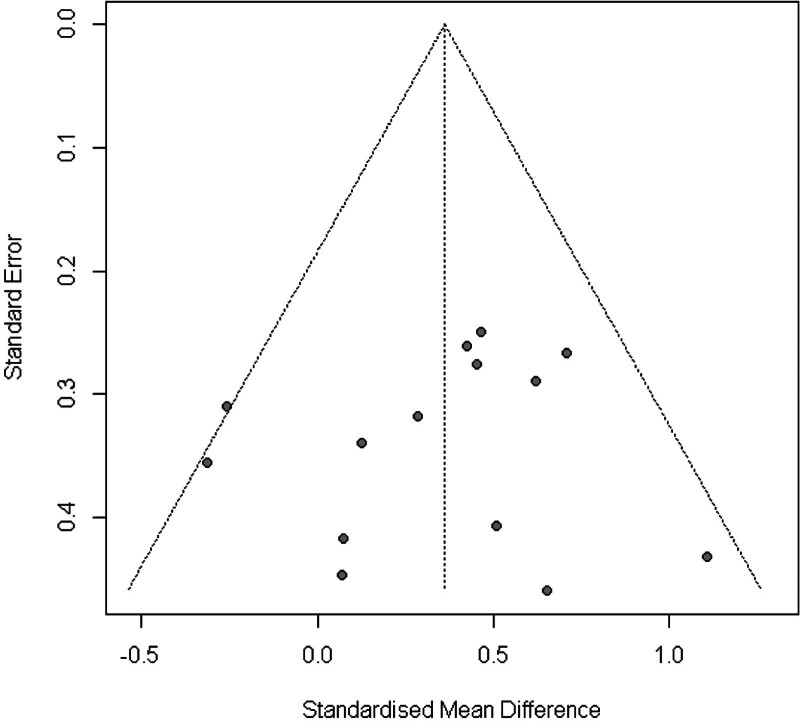
Funnel plot of WB-EMS studies addressing body fat. WB-EMS = whole-body electromyostimulation.

Figure 5 shows the Funnel plot of the WB-EMS on the effects of total body fat that provided no evidence of significant bias.^[61]^ Egger regression test^[31]^ for funnel plot asymmetry did not indicate significant asymmetry (*P* = .7).

### 3.7. WB-EMS effects on maximal strength

**Figure 6.**
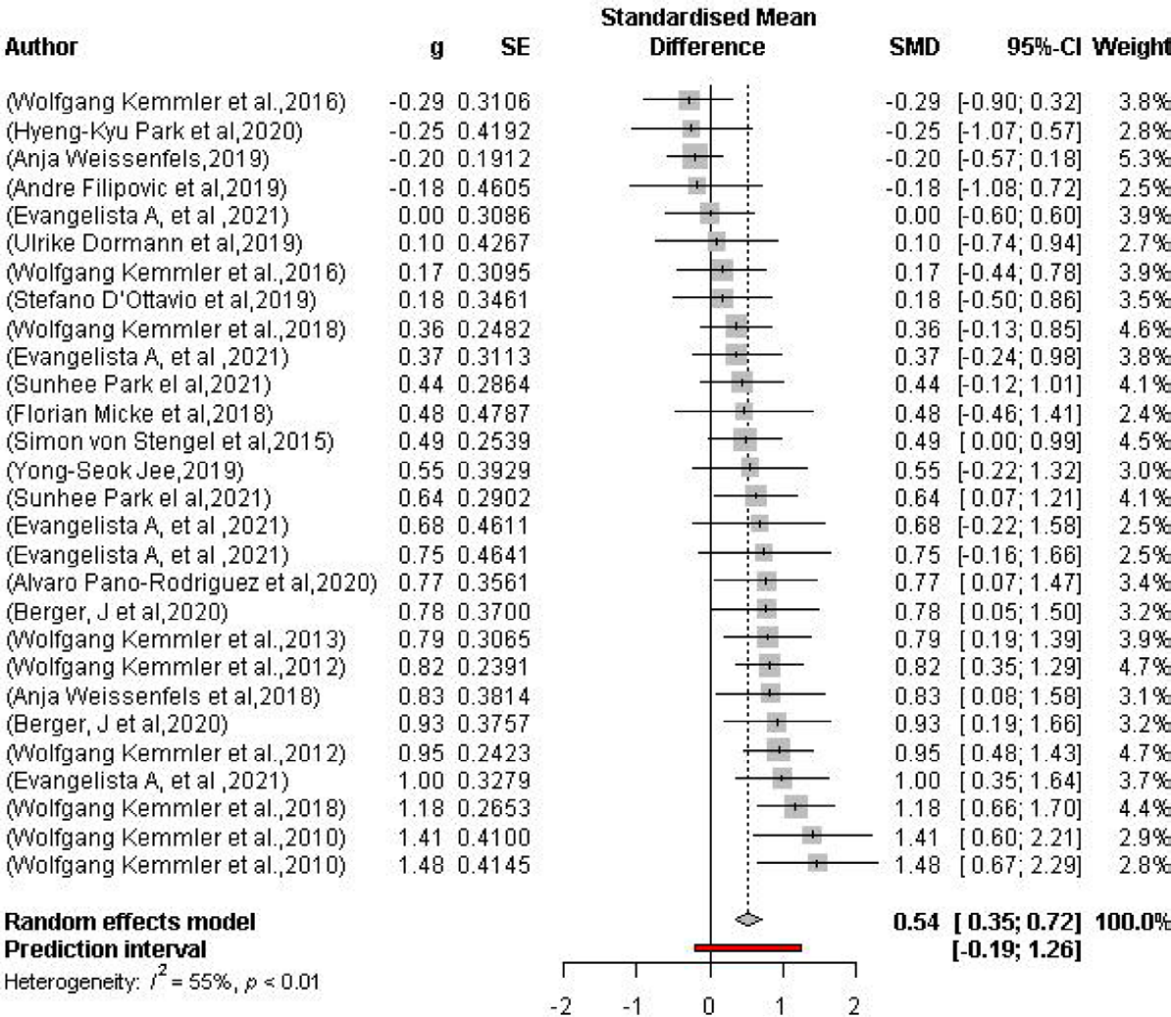
Forest plot of the results of the meta-analysis on strength. Data are shown as pooled standard mean differences (SMD) with 95% CI for changes in the WB-EMS and non-EMS training groups. CI = confidence interval, WB-EMS = whole-body electromyostimulation.

Twenty-eight maximal strength variables (knee extension, handgrip, trunk flexion, and extension strength) from 19 studies determined the effect of whole body electromyostimulation on maximal strength (Fig. 6). In summary, WB-EMS very significantly (*P* < .0001) affected strength. The pooled estimate from random effects analysis was SMD 0.54 95% CI: 0.35–0.72. A moderate level of heterogeneity was observed between trials (*I*^2^ = 55%, *P* = .02).

**Figure 7.**
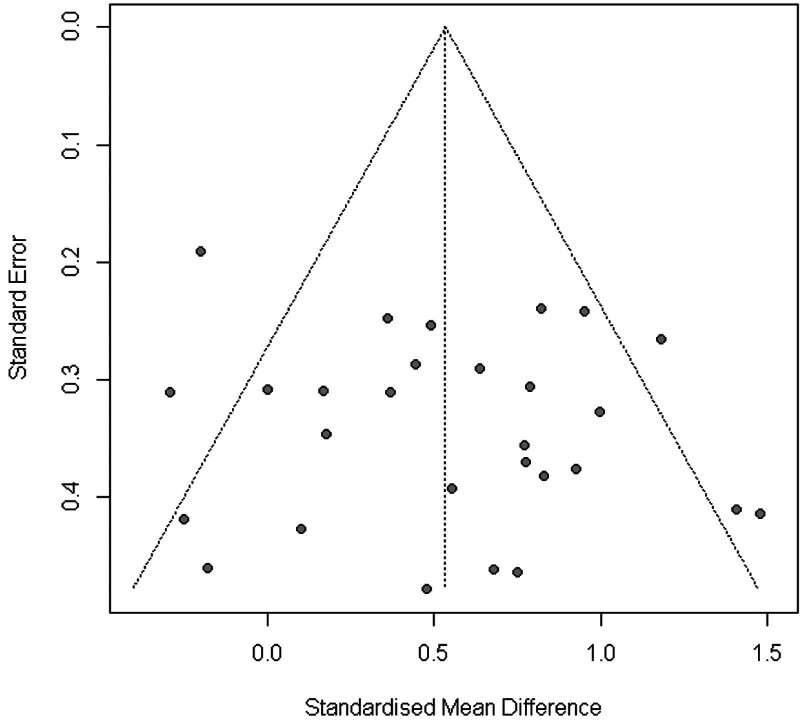
Funnel plot of WB-EMS studies on maximal strength. WB-EMS = whole-body electromyostimulation.

Figure 7 shows the Funnel plot of the WB-EMS on the effects of total body fat that provided no evidence of significant bias.^[61]^ Egger regression test^[31]^ for funnel plot asymmetry did not indicate significant asymmetry (*P* = .3).

### 3.8. Effects of EEG on muscle power

**Figure 8.**
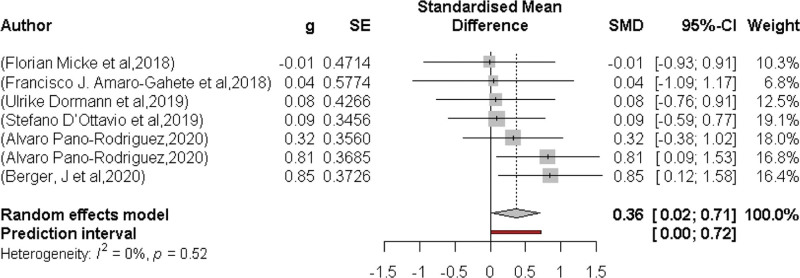
Forest plot of the results of the meta-analysis on muscle power. Data are shown as pooled standardized mean differences (SMD) with 95% CI for changes in the WB-EMS and control groups. CI = confidence interval, WB-EMS = whole-body electromyostimulation.

For muscular power, a total of 7 works determined the effect of the WB-EMS (Fig. 8). Whole body electromyostimulation affected muscle power significantly (*P* = .04). The standard mean difference was SMD 0.36 95% CI: 0.02–0.71. A very low level of heterogeneity was observed between trials (*I*^2^ = 0%, *P* = .52).

**Figure 9.**
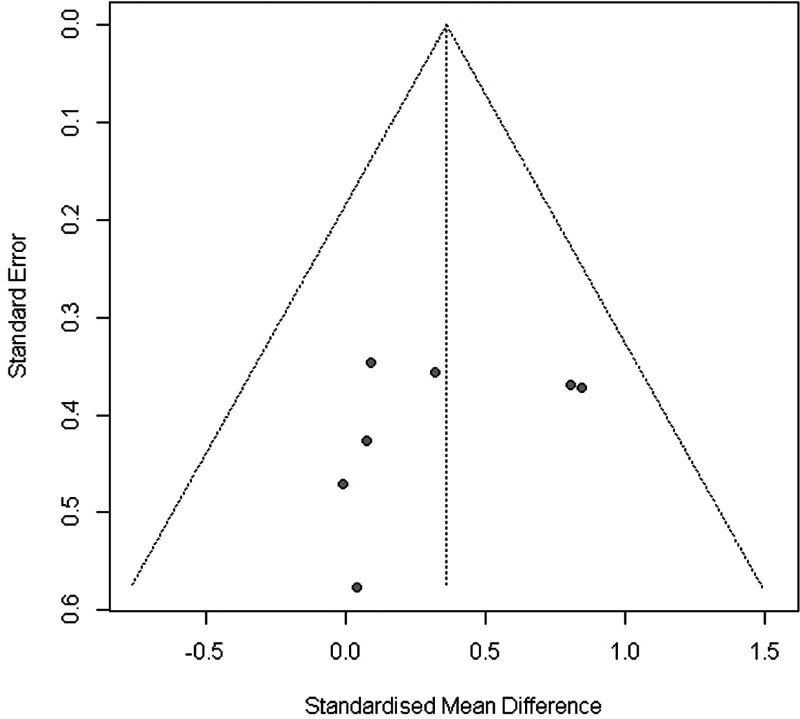
Funnel plot of WB-EMS studies addressing muscle power. WB-EMS = whole-body electromyostimulation.

Figure 9 shows the Funnel plot of the WB-EMS on the effects of total body fat that provided no evidence of significant bias.^[61]^ Egger regression test^[31]^ for funnel plot asymmetry did not indicate significant asymmetry (*P* = .4).

## 4. Discussion

The existing literature is not unanimous on the effects of WB-EMS training on body composition. Mainly on body fat reduction, there are studies reporting significant differences for the WB-EMS group^[16,52,56]^ and others in which its effects were not significant.^[42,62]^

This heterogeneity, to a large extent, can be explained by 4 main factors: application of different types of intensity (from low to high intensity); different training volumes (from 20 to 90 minutes per week, from 6 weeks to 12 months of intervention); little or no control over participants’ caloric intake in most studies; and different population types (WB-EMS, trained participants, healthy, obese, cancer patients, and others).

This heterogeneity in the literature is again evidenced by our review work with meta-analysis, where our findings indicated that WB-EMS significantly affected participants’ decrease in body fat, which differs from that reported in another review conducted in 2021 on the effects of WB-EMS on body composition and strength in untrained.^[24]^ In this case, this difference can possibly be explained by the fact our study included a larger number of studies because it is more current. Nor have we differentiated between trained and untrained populations.

On the other hand, there is more consensus in the literature regarding alterations in muscle mass. Several studies report a positive effect with significant differences for the increase in muscle mass for the WB-EMS training group.^[26,37,39,46]^ In our review, in which we have included 14 articles, the WB-EMS had significant effects (*P* = 000.2) with the SMD (0.36; 95% CI: 0.16–0.57). In the 2021 revision by Wolfgang Kemmler,^[24]^ the results are even more significant (1.23; 95% CI: 0.71–1.76) for the intervention group in a total of 13 groups analyzed. In addition, WB-EMS appears to be very effective in maintaining muscle mass in the process of weight loss (calorie restriction), as demonstrated by these 2 studies.^[45,63]^

There is some evidence of a dose/response effect of pulse intensity that affects body fat more than muscle mass.^[41]^ Few studies^[45,62]^ have taken into account different energy intakes (restriction from 250 to 500 kcal), while others have supplemented participants with different doses of whey protein,^[26,46,64,65]^ nutritional conditions decisive for weight loss (negative energy balance) and for muscle mass gain, respectively (minimum adequate protein intake).^[40,66]^ Regarding the type of sessions, most of the studies conducted and included in the reviews have used the same programs and types of exercises, being used for both objectives. In other words, there was no specific approach for each training objective. These factors may go some way to explain the less obvious effects on body fat loss, which contrasts with the greater evidence for effects on muscle mass. In summary, more studies of high methodological quality are still needed to determine the scientific evidence for the use of WB-EMS for body fat loss. Although the factors that determine strength production are several, we know that the amount of muscle mass (cross-sectional area) is one of the main ones.^[67]^ Thus, the effectiveness of WB-EMS in increasing muscle mass also seems to be accompanied by an increase in maximal strength. In the review by Wolgang Kemmler (2021), significant effects on maximal extension and trunk extension strength were observed, with a total of 10 papers included in the meta-analysis. This result is in agreement with our systematic review and meta-analysis, where we found statistically significant differences for the intervention group (*P* < .0001), with a SMD of 0.54; 95% CI: 0.35–0.92, in a total of 19 included papers and 28 analyzed groups with different muscle groups evaluated.

In contrast, we also analyzed muscle power, where we also found statistically significant differences in favor of the intervention group in a total of 7 articles, although with a lower degree of significance (*P* = .036) and smaller effect (0.36; CI 95%:0.01–0.70). These results are, in part, inconsistent with a mini-review conducted with 5 studies and 112 participants, where the authors found statistically significant differences for maximal strength, but not for power.^[68]^ It should be noted that this mini-review was conducted with moderately trained young adults, unlike ours, where we included all types of populations.

### 4.1. Limitations

The findings of the study should be considered in the context of the following limitations:

• The criteria used to judge the level of evidence have not yet been standardized. Different authors of systematic reviews use different criteria, and the same author may use different criteria in different studies.^[69]^
• The use of different criteria is related to the decision to include only randomized clinical trials or to also consider studies of low methodological quality, where the measurement scales may also vary.^[70]^

The best method for assessing the risk of bias has not been determined. The search strategy only looked for articles in English, which implied a risk of bias, as publishing significant results is easier than publishing non-significant results, and the latter is more likely to appear in national journals written in languages other than English.^[71]^

## 5. Conclusion

This systematic review and meta-analysis provided further evidence of the positive effects of WB-EMS on body composition and strength parameters. The hypotheses we defined for this work have been confirmed. WB-EMS showed a significant effect on muscle mass and on the reduction of body fat. The effects on the secondary hypotheses were also confirmed with a significant effect on maximal strength and muscular power.

Being a sample with these characteristics, extrapolation of the results should only be made to cohorts in terms of age (middle age or older) and level of physical activity (untrained or less trained). Furthermore, it should be considered that the present results can only be attributed to WB-EMS protocols that focus on moderate to high impulse intensity and low to moderate voluntary workload (functional bodyweight exercises).

Further systematic reviews and meta-analyses with more studies in young and healthy populations, will be necessary for further evidence and generalization of the results obtained with this work.

## Author contributions

**Conceptualization:** Luiz Rodrigues-Santana, Louro Hugo, Miguel A. Hernández-Mocholí, José C. Adsuar.

**Data curation:** Miguel A. Hernández-Mocholí, Nicolás Contreras-Barraza.

**Funding acquisition:** José C. Adsuar.

**Methodology:** Nicolás Contreras-Barraza.

**Software:** Miguel A. Hernández-Mocholí.

**Writing – original draft:** Luiz Rodrigues-Santana, Jorge Pérez-Gómez, Miguel A. Hernández-Mocholí, Jorge Carlos-Vivas, Pilar Saldaña-Cortés, Nicolás Contreras-Barraza, José C. Adsuar.

**Writing – review & editing:** Luiz Rodrigues-Santana, Jorge Pérez-Gómez, Miguel A. Hernández-Mocholí, Jorge Carlos-Vivas, Pilar Saldaña-Cortés, Nicolás Contreras-Barraza, José C. Adsuar.

## References

[1] Garber CE Blissmer B Deschenes MR. Quantity and quality of exercise for developing and maintaining cardiorespiratory, musculoskeletal, and neuromotor fitness in apparently healthy adults: guidance for prescribing exercise. Med Sci Sports Exerc. 2011;43:1334–59. 21694556 10.1249/MSS.0b013e318213fefb

[2] Wewege M van den Berg R Ward RE. The effects of high-intensity interval training vs. moderate-intensity continuous training on body composition in overweight and obese adults: a systematic review and meta-analysis. Obes Rev. 2017;18:635–46. 28401638 10.1111/obr.12532

[3] Santanasto AJ Goodpaster BH Kritchevsky SB. Body composition remodeling and mortality: the health aging and body composition study. J Gerontol Ser A Biol Sci Med Sci. 2017;72:513–9. 27567109 10.1093/gerona/glw163 PMC5897837

[4] Percent body fat norms for men and women. Available at: https://www.acefitness.org/education-and-resources/lifestyle/tools-calculators/percent-body-fat-calculator/ [access date December 7, 2021]

[5] Peterson MD Sen A Gordon PM. Influence of resistance exercise on lean body mass in aging adults: a meta-analysis. Med Sci Sports Exerc. 2011;43:249–58. 20543750 10.1249/MSS.0b013e3181eb6265 PMC2995836

[6] Michell V Samaria C Junior Rudy N. Effects of a concurrent physical exercise program on aerobic power and body composition in adults. J Sports Med Phys Fitness. 2014;54:441–6. 25034548

[7] Nybo L Sundstrup E Jakobsen MD. High-intensity training versus traditional exercise interventions for promoting health. Med Sci Sports Exerc. 2010;42:1951–8. 20195181 10.1249/MSS.0b013e3181d99203

[8] Kemmler W Teschler M Weissenfels A. Whole-body electromyostimulation versus high intensity (resistance exercise) training – Impact on body composition and strength. Dtsch Z Sportmed. 2015;66:321–7.

[9] de la Camara Serrano MA Pardos Sevilla AI. Review of the physical benefits of whole-body electromyostimulation. Apunts Educacion Fisica Y Deportes. 2016:28–33.

[10] Amaro-Gahete FJ De-la-O A Jurado-Fasoli L. Changes in physical fitness after 12 weeks of structured concurrent exercise training, high intensity interval training, or whole-body electromyostimulation training in sedentary middle-aged adults: a randomized controlled trial. Front Physiol. 2019;10:451. 31105580 10.3389/fphys.2019.00451 PMC6492765

[11] Choi G Hyon P Song J. Effects of the micro-training with EMS device on body composition, isokinetic muscular function, and physical fitness of healthy 20’s males. Korean Soc Sports Sci. 2016;25:1143–54.

[12] Filipovic A Grau M Kleinöder H. Effects of a whole-body electrostimulation program on strength, sprinting, jumping, and kicking capacity in elite soccer players. J Sports Sci Med. 2016;15:639–48. 27928210 PMC5131218

[13] Filipovic A DeMarees M Grau M. Superimposed whole-body electrostimulation augments strength adaptations and type II myofiber growth in soccer players during a competitive season. Front Physiol. 2019;10:1187. 31607944 10.3389/fphys.2019.01187 PMC6768094

[14] Schuhbeck E Birkenmaier C Schulte-Goecking H. The influence of WB-EMS-training on the performance of ice hockey players of different competitive status. Front Physiol. 2019;10:1136. 31551812 10.3389/fphys.2019.01136 PMC6746827

[15] Amaro-Gahete FJ Alejandro D-l-O Sanchez-Delgado G. Whole-body electromyostimulation improves performance-related parameters in runners. Front Physiol. 2018;9. 10.3389/fphys.2018.01576 PMC6242945 30483147

[16] Kemmler W Birlauf A von S. Effects of whole-body-electromyostimulation on body composition and cardiac risk factors in elderly men with the metabolic syndrome. The Test-II study. Dtsch Z Sportmed. 2010;61:117–23.

[17] Kemmler W Bebenek M Engelke K. Impact of whole-body electromyostimulation on body composition in elderly women at risk for sarcopenia: the Training and ElectroStimulation Trial (TEST-III). Age. 2014;36:395–406. 23949160 10.1007/s11357-013-9575-2 PMC3889893

[18] Kemmler W von Stengel S Teschler M. Whole-body electromyostimulation and sarcopenic obesity results of the randomized controlled FORMOsA - Sarcopenic obesity study. Osteologie. 2016;25:204–11. 10.1007/s00198-016-3662-z 27289534

[19] Martinez-Amat A Aibar-Almazan A Fabrega-Cuadros R. Exercise alone or combined with dietary supplements for sarcopenic obesity in community-dwelling older people: a systematic review of randomized controlled trials. Maturitas. 2018;110:92–103. 29563041 10.1016/j.maturitas.2018.02.005

[20] von Stengel S Bebenek M Engelke K. Whole-body electromyostimulation to fight osteopenia in elderly females: the randomized controlled Training and Electrostimulation Trial (TEST-III). J Osteoporos. 2015;2015:643520. 25785225 10.1155/2015/643520 PMC4345062

[21] Amaro-Gahete FJ De la O A Robles-Gonzalez L. Impact of two whole-body electromyostimulation training modalities on body composition in recreational runners during endurance training cessation. Ricyde-Revista Internacional De Ciencias Del Deporte. 2018;14:205–18.

[22] Evangelista AL Teixeira CVL Barros BM. Does whole-body electrical muscle stimulation combined with strength training promote morphofunctional alterations? Clinics. 2019;7:74. 10.6061/clinics/2019/e1334 PMC6820510 31721936

[23] Rodrigues-Santana L Louro H Denche-Zamorano A. Profile of whole body electromyostimulation training users-A pilot study. Int J Environ Res Public Health. 2022;19. 10.3390/ijerph19084711 PMC9029882 35457575

[24] Kemmler W Shojaa M Steele J. Efficacy of whole-body electromyostimulation (WB-EMS) on body composition and muscle strength in non-athletic adults. A systematic review and meta-analysis. Front Physiol. 2021;12:640657. 33716787 10.3389/fphys.2021.640657 PMC7952886

[25] Liberati A Altman DG Tetzlaff J. The PRISMA statement for reporting systematic reviews and meta-analyses of studies that evaluate health care interventions: explanation and elaboration. PLoS Med. 2009;6:e1000100. 19621070 10.1371/journal.pmed.1000100 PMC2707010

[26] Kemmler W Weissenfels A Teschler M. Whole-body electromyostimulation and protein supplementation favorably affect sarcopenic obesity in community-dwelling older men at risk: the randomized controlled FranSO study. Clin Interv Aging. 2017;12:1503–13. 28989278 10.2147/CIA.S137987 PMC5624743

[27] Maher CG Sherrington C Herbert RD. Reliability of the PEDro scale for rating quality of randomized controlled trials. Phys Ther. 2003;83:713–21. 12882612

[28] Schunemann HJ Oxman AD Brozek J. GRADE: grading quality of evidence and strength of recommendations for diagnostic tests and strategies. BMJ-Brit Med J. 2008;336:1106–10. 10.1136/bmj.39500.677199.AE PMC2386626 18483053

[29] Lüdecke D. esc: Effect Size Computation for Meta Analysis (Version 0.5.1). 2018;1. doi: 10.5281/zenodo.1249218, https://CRAN.R-project.org/package=esc.

[30] Higgins JPT Thompson SG Deeks JJ. Measuring inconsistency in meta-analyses. Brit Med J. 2003;327:557–60. 12958120 10.1136/bmj.327.7414.557 PMC192859

[31] Egger M Smith GD Schneider M. Bias in meta-analysis detected by a simple, graphical test. BMJ Brit Med J. 1997;315:629–34. 9310563 10.1136/bmj.315.7109.629 PMC2127453

[32] Viechtbauer W. Conducting meta-analyses in R with the metafor package. J Stat Softw. 2010;36:1–48.

[33] Higgins JPT Thomas J Chandler J., eds. Cochrane Handbook for Systematic Reviews of Interventions. 2nd ed. Chichester (UK): John Wiley & Sons; 2019.

[34] Weissenfels A Wirtz N Doermann U. Comparison of whole-body electromyostimulation versus recognized back-strengthening exercise training on chronic nonspecific low back pain: a randomized controlled study. Biomed Res Int. 2019;2019:5745409. 31687394 10.1155/2019/5745409 PMC6794965

[35] Doermann U Wirtz N Micke F. The effects of superimposed whole-body electromyostimulation during short-term strength training on physical fitness in physically active females: a randomized controlled trial. Front Physiol. 2019;10:728. 31316389 10.3389/fphys.2019.00728 PMC6610316

[36] Wittmann K Sieber C von Stengel S. Impact of whole body electromyostimulation on cardiometabolic risk factors in older women with sarcopenic obesity: the randomized controlled FORMOsA-sarcopenic obesity study. Clin Interv Aging. 2016;11:1697–706. 27920508 10.2147/CIA.S116430 PMC5123721

[37] Kemmler W von Stengel S. Whole-body electromyostimulation as a means to impact muscle mass and abdominal body fat in lean, sedentary, older female adults: subanalysis of the TEST-III trial. Clin Interv Aging. 2013;8:1353–64. 24130433 10.2147/CIA.S52337 PMC3795534

[38] Park S Min S Park SH. Influence of isometric exercise combined with electromyostimulation on inflammatory cytokine levels, muscle strength, and knee joint function in elderly women with early knee osteoarthritis. Front Physiol. 2021;12:688260. 34326779 10.3389/fphys.2021.688260 PMC8313868

[39] Kim J Jee Y. EMS-effect of exercises with music on fatness and biomarkers of obese elderly women. Medicina-Lithuania. 2020;56:158. 10.3390/medicina56040158 PMC7231244 32244777

[40] Kim JY. Optimal diet strategies for weight loss and weigh loss maintenance. J Obes Metab Syndr. 2021;30:20–31. 33107442 10.7570/jomes20065 PMC8017325

[41] Jee Y-S. The effect of high-impulse-electromyostimulation on adipokine profiles, body composition and strength: a pilot study. Isokinet Exerc Sci. 2019;27:163–76.

[42] Park H-K Na SM Choi S-L. Physiological effect of exercise training with whole body electric muscle stimulation suit on strength and balance in young women: a randomized controlled trial. Chonnam Med J. 2021;57:76–86. 33537223 10.4068/cmj.2021.57.1.76 PMC7840343

[43] Pano-Rodriguez A Vicente Beltran-Garrido J Hernandez-Gonzalez V. Impact of whole body electromyostimulation on velocity, power and body composition in postmenopausal women: a randomized controlled trial. Int J Environ Res Public Health. 2020;10:17. 10.3390/ijerph17144982 PMC7400631 32664361

[44] Pano-Rodriguez A Vicente Beltran-Garrido J Hernandez-Gonzalez V. Effects of whole-body electromyostimulation on physical fitness in postmenopausal women: a randomized controlled trial. Sensors. 2020;8:20. 10.3390/s20051482 PMC7085547 32182674

[45] Willert S Weissenfels A Kohl M. Effects of whole-body electromyostimulation on the energy-restriction-induced reduction of muscle mass during intended weight loss. Front Physiol. 2019;10:1012. 31456693 10.3389/fphys.2019.01012 PMC6699561

[46] Kemmler W Grimm A Bebenek M. Effects of combined whole-body electromyostimulation and protein supplementation on local and overall muscle/fat distribution in older men with sarcopenic obesity: the randomized controlled Franconia Sarcopenic Obesity (FranSO) Study. Calcif Tissue Int. 2018;103:266–77. 29675640 10.1007/s00223-018-0424-2

[47] Kemmler W Kohl M Stengel S. Effects of high intensity resistance training versus whole-body electromyostimulation on cardio-metabolic risk factors in untrained middle aged males. a randomized controlled trial contribution/ originality. J Sports Res. 2016;2016:44–55.

[48] Micke F Weissenfels A Wirtz N. Similar pain intensity reductions and trunk strength improvements following whole-body electromyostimulation vs. whole-body vibration vs. conventional back-strengthening training in chronic non-specific low back pain patients: a three-armed randomized controlled trial. Front Physiol. 2021;12:664991. 33927646 10.3389/fphys.2021.664991 PMC8076746

[49] Evangelista AL Alonso AC Ritti-Dias RM. Effects of whole body electrostimulation associated with body weight training on functional capacity and body composition in inactive older people. Front Physiol. 2021;12:638936. 33927638 10.3389/fphys.2021.638936 PMC8078052

[50] Zink-Ruckel C Chaudry O Engelke K. Once weekly whole-body electromyostimulation enhances muscle quality in men: data of the randomized controlled franconian electromyostimulation and golf study. Front Physiol. 2021;20:11. 10.3389/fphys.2021.700423 PMC8335588 34366890

[51] Weissenfels A Teschler M Willert S. Effects of whole-body electromyostimulation on chronic nonspecific low back pain in adults: a randomized controlled study. J Pain Res. 2018;11:1949–57. 30288089 10.2147/JPR.S164904 PMC6160275

[52] Kemmler W Teschler M Weissenfels A. Whole-body EMS to fight sarcopenic obesity? A review with emphasis on body fat. Deutsche Zeitschrift für Sportmedizin. 2017;68:170–7.

[53] Kemmler W Schliffka R Mayhew JL. Effects of whole-body electromyostimulation on resting metabolic rate, body composition, and maximum strength in postmenopausal women: the training and electrostimulation trial. J Strength Cond Res. 2010;24:1880–7. 20555279 10.1519/JSC.0b013e3181ddaeee

[54] D’Ottavio S Briotti G Rosazza C. Effects of two modalities of whole-body electrostimulation programs and resistance circuit training on strength and power. Int J Sports Med. 2019;40:831–41. 31533156 10.1055/a-0982-3311

[55] Berger J Becker S Ludwig O. Whole-body electromyostimulation in physical therapy: do gender, skinfold thickness or body composition influence maximum intensity tolerance? J Phys Ther Sci. 2020;32:395–400. 32581432 10.1589/jpts.32.395 PMC7276779

[56] Kemmler W Teschler M Weissenfels A. Effects of whole-body electromyostimulation versus high-intensity resistance exercise on body composition and strength: a randomized controlled study. Evid Based Complement Altern Med. 2016;2016:9236809. 10.1155/2016/9236809 PMC4789460 27034699

[57] Borg G. Ratings of perceived exertion and heart-rates during short-term cycle exercise and their use in a new cycling strength test. Int J Sports Med. 1982;3:153–8. 7129724 10.1055/s-2008-1026080

[58] Micke F Kleinoeder H Doermann U. Effects of an eight-week superimposed submaximal dynamic whole-body electromyostimulation training on strength and power parameters of the leg muscles: a randomized controlled intervention study. Front Physiol. 2018;5:9. 10.3389/fphys.2018.01719 PMC6290057 30568596

[59] Guyatt GH Oxman AD Vist GE. GRADE: an emerging consensus on rating quality of evidence and strength of recommendations. BMJ. 2008;336:924–6. 18436948 10.1136/bmj.39489.470347.AD PMC2335261

[60] Balshem H Helfand M Schünemann HJ. GRADE guidelines: 3. Rating the quality of evidence. J Clin Epidemiol. 2011;64:401–6. 21208779 10.1016/j.jclinepi.2010.07.015

[61] Sterne JAC Sutton AJ Ioannidis JPA. Recommendations for examining and interpreting funnel plot asymmetry in meta-analyses of randomised controlled trials. BMJ Brit Med J. 2011;343. 10.1136/bmj.d4002 21784880

[62] Ricci PA Di Thommazo-Luporini L Jurgensen SP. Effects of whole-body electromyostimulation associated with dynamic exercise on functional capacity and heart rate variability after bariatric surgery: a randomized, double-blind, and sham-controlled trial. Obes Surg. 2020;30:3862–71. 32447638 10.1007/s11695-020-04724-9

[63] Bellia A Ruscello B Bolognino R. Whole-body electromyostimulation plus caloric restriction in metabolic syndrome. Int J Sports Med. 2020;41:751–8. 32485778 10.1055/a-1171-2003

[64] Reljic D Herrmann HJ Neurath MF. High-protein nutrition and whole-body electromyostimulation in hematologic-oncological patients: is a new option? Internist (Berl). 2018;59:S29–30.

[65] Schink K Herrmann HJ Schwappacher R. Whole-body electromyostimulation combined with personalized nutritional support improves the body composition of patients with advanced cancer. Internist (Berl). 2018;59:S67-S.

[66] Joanisse S McKendry J Lim C. Understanding the effects of nutrition and post-exercise nutrition on skeletal muscle protein turnover: insights from stable isotope studies. Clin Nutr Open Sci. 2021;36:56–77.

[67] Reggiani C Schiaffino S. Muscle hypertrophy and muscle strength: dependent or independent variables? A provocative review. Eur J Transl Myology. 2020;30. 10.4081/ejtm.2020.9311 PMC7582410 33117512

[68] Wirtz N Doermann U Micke F. Effects of whole-body electromyostimulation on strength-, sprint-, and jump performance in moderately trained young adults: a mini-meta-analysis of five homogenous RCTs of our work group. Front Physiol. 2019;10. 10.3389/fphys.2019.01336 PMC6857204 31780950

[69] Ferreira PH Ferreira ML Maher CG. Effect of applying different “levels of evidence” criteria on conclusions of Cochrane reviews of interventions for low back pain. J Clin Epidemiol. 2002;55:1126–9. 12507677 10.1016/s0895-4356(02)00498-5

[70] del Pozo-Cruz B Adsuar JC Parraca JA. Using whole-body vibration training in patients affected with common neurological diseases: a systematic literature review. J Altern Complement Med. 2012;18:29–41. 22233167 10.1089/acm.2010.0691

[71] Furlan AD Pennick V Bombardier C. 2009 updated method guidelines for systematic reviews in the cochrane back review group. Spine. 2009;34:1929–41. 19680101 10.1097/BRS.0b013e3181b1c99f

